# *Dodonaea viscosa* var. *angustifolia* Inhibits Germ Tube and Biofilm Formation by *C. albicans*

**DOI:** 10.1155/2013/261978

**Published:** 2013-10-09

**Authors:** Serisha Devi Naicker, Mrudula Patel

**Affiliations:** Division of Oral Microbiology, Department of Clinical Microbiology and Infectious Diseases, National Health Laboratory Services and Faculty of Health Sciences, University of the Witwatersrand, Private Bag 3, Wits, Johannesburg 2050, Gauteng, South Africa

## Abstract

The virulence factors of *Candida albicans* are germ tube and biofilm formation, adherence to host tissues, and production of hydrolytic enzymes. This study investigated the effect of *Dodonaea viscosa *var. * angustifolia* extract on the germ tube and biofilm formation of *C. albicans*. Serum containing the three subinhibitory concentrations of leaf extract was inoculated with *C. albicans*, incubated, and viewed under a light microscope. Number of cells with germ tube was recorded and the results were analysed using Scheffe test for pairwise comparison. Biofilms were grown on coverslips in the presence of plant extracts and processed for scanning electron microscopy (SEM). Planktonic cells were grown in the presence of plant extract for 6 h and processed for electron microscopy (TEM). The crude plant extract significantly (*P* < 0.01) reduced the germ tube formation of *C. albicans* at 3.125 (85.36%), 1.56 (61.91%), and 0.78 mg/mL (26.27%) showing a concentration dependent effect. SEM results showed concentration dependent reduction in biofilm and hyphae formation. TEM results showed that the plant extract caused damage to the cell wall and cell membrane. DVA extract has ability to reduce virulence of *C. albicans* by inhibiting germ tube and biofilm formation through damage to the cell wall. Therefore, it has therapeutic potential.

## 1. Introduction

Oral candidiasis is an opportunistic infection of the oral cavity which usually occurs in the elderly and immunocompromised individuals [1]. *Candida albicans* is the most common species of yeast isolated from patients with oral candidiasis [2]. It can cause superficial infections such as thrush and denture stomatitis; however in more severe cases, it causes life-threatening systemic mycoses. Most of these commensal *C. albicans* cells proliferate as budding yeasts. However, when the epithelial barrier is breached due to trauma, immunosuppression, or hormonal alterations, the budding form is converted into a hyphal form which causes invasion of submucosal tissues [3]. The ability to switch between yeast and hyphal morphologies can play a role in the virulence of *C. albicans *[4]. Other virulence properties are adherence to host tissues and prostheses and ability to produce hydrolytic enzymes. Some of these factors are interrelated. *C. albicans* cells bearing germ tubes are more adherent to buccal epithelial cells (BEC) than yeast forms of *C. albicans *[5] due to the antigens found on germ tubes [6]. 

Many effective antifungal agents such as amphotericin B, nystatin, and fluconazole are available. However, poor solubility, stability, and absorption, toxicity, and the development of drug resistance are a problem with existing drugs [7]. A novel approach to therapy has been suggested by some researchers, where the virulence of *C. albicans* can be targeted instead of an antimicrobial property. This would be ideal where the causative organism is a part of the normal flora, such as *C. albicans* in the oral cavity [2, 8]. Pathogenic characteristics such as germ tube and biofilm formation and production of tissue damaging enzymes are possible targets of new drugs. 

Many medicinal plants used in Africa have been explored for their anticandida activities [9]. *D. viscosa *var*. angustifolia* (DVA) belongs to the Sapindaceae family and it is found in many parts of the world including South Africa. Leaves and tips of the twigs have many medicinal properties and they are traditionally used to treat colds, fever, flu, sore throats, and oral thrush worldwide [10, 11]. It has demonstrated in vitro anticandida activity at high (MIC of 6.25–25 mg/mL) concentrations [12]. The present study investigated the effect of subinhibitory concentration of crude extract of DVA on the germ tube and biofilm formation by *C. albicans*.

## 2. Materials and Methods

### 2.1. Cultures

Three *C. albicans* strains, two isolated from the oral cavities of HIV positive patients and an ATCC 90028 strain, were used in the study. Ethical clearance was obtained from the Committee for Research on Human Subjects (Medical), University of the Witwatersrand, Johannesburg (Certificate number M000402). Written consent was obtained from the subjects. Strains, were cultured onto Sabouraud dextrose agar and identified to species level using the germ tube technique and the API 20 C sugar assimilation tests. *C. albicans* suspensions were prepared with an optical density of 0.3 (405 nm) which equates to approximately 10^6^–10^7^ cells/mL. This solution was used as an inoculum for the experiments.

### 2.2. Plant Material and Extract Preparation

Plant material was collected in January 2011 from the Pypeklipberg, Mkhunyane Eco Reserve, Mpumalanga province of South Africa. Previously the plant was verified as family Sapindaceae, *Dodonaea viscosa *var*. angustifolia* Benth by a taxonomist, from the Herbarium at the University of the Witwatersrand. Voucher specimens number J 94882 were deposited in this Herbarium. Leaves were dried under shade and then milled to a fine powder. Acetone extracts were prepared as described by Patel and Coogan in 2008 [12]. As required, the crude dry extract was weighed and dissolved in acetone to obtain a concentration of 50 mg of crude extract per mL of solvent. Fresh plant extracts were prepared for each experiment. Three subinhibitory concentrations of 3.125, 1.56, and 0.78 mg/mL were selected based on the MIC results reported by Patel and Coogan (2008) for the subsequent experiments [12]. 

### 2.3. Effect on the Germ Tube Formation

Effect of the crude plant extract on the germ tube formation was studied using a technique described by Mackenzie (1962) with modification [13]. Briefly, 2 mL of sterile horse serum containing the 3 subinhibitory concentrations (3.125, 1.56, and 0.78 mg/mL) of crude plant extract was inoculated with 10 *μ*L of culture inoculum. Horse serum without plant extract was included as a control. The tubes were incubated for 3 h at 37°C. Smears of each culture were prepared, heat-fixed, and stained with crystal violet for 1 minute. Per smear, fifty cells were randomly selected and the number of cells with germ tube was recorded. Cells were considered germinated if they had a germ tube at least twice the length of the cell. These experiments were repeated three times. The number of yeast cells with germ tube exposed to the various test concentrations was compared to that of the unexposed controls using an ANOVA and the Scheffe test.

### 2.4. Effect on the Biofilm Formation

The effect of the crude plant extract on biofilm formation was studied using a technique described by Bandara et al. (2010) with some modifications [14]. Four Thermanox plastic cover slips with 25 mm diameter were coated with 1 mL filter sterilized human saliva in 6 well tissue culture plates at 37°C for 1 hour. The saliva was removed and a 1 mL of *C. albicans* inoculums was placed into each well covering the cover slips for 3 hours. The nonadherent cells were removed by washing the cover slips with sterile distilled water. Three cover slips were exposed to Sabouraud dextrose broth containing 3 subinhibitory concentrations (3.125, 1.56, and 0.78 mg/mL) of plant extract for a week. The fourth cover slip was covered with Sabouraud dextrose broth only and was used as an unexposed control. On alternate days, the medium with and without the plant extract was changed. The cover slips were washed with PBS, fixed with 2.5% glutaraldehyde overnight at 4°C, washed again with PBS, and dehydrated in a series of ethanol washes. The cover slips underwent critical point drying and were mounted onto aluminium stubs and viewed under the Scanning Electron Microscope (Joel 840S, Joel Ltd, Tokyo). These experiments were repeated three times.

### 2.5. Transmission Electron Microscopy (TEM) of Planktonic Yeast Cells

Ten milliliters of Sabouraud dextrose broth containing the subinhibitory concentrations (3.125, 1.56, and 0.78 mg/mL) of plant extract was inoculated with 1 mL of inoculums and incubated at 37°C while shaking at 60 rpm for 6 hours. Water was used as a control. Yeast cells were then harvested, washed 3 times with phosphate buffered saline (PBS) at pH 7.3, and fixed in a solution of 2.5% glutaraldehyde overnight at 4°C. After fixation, yeasts were washed 3 times in PBS pH 7.3 and postfixed for 2 h in 1% osmium tetroxide at 4°C. Cells were washed again with PBS at pH 7.3, dehydrated in successive ethanol washes (50%, 70%, 80%, 95%, and 100%), and embedded in Spurrs resin. Ultrathin sections were cut, stained with uranyl acetate and lead citrate [15], and viewed under TEM (JEM 100S).

## 3. Results

### 3.1. Germ Tube Formation

**Figure 1:**
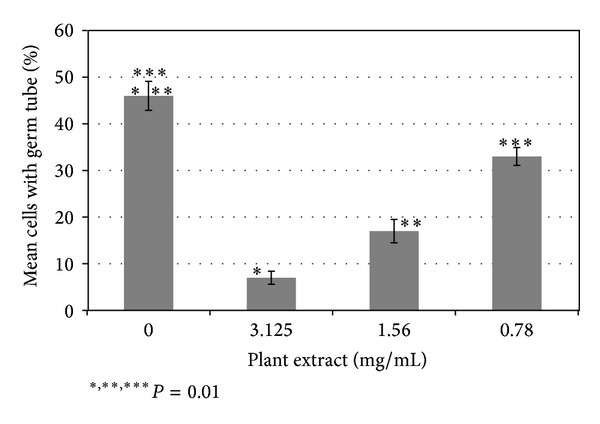
The effect of *Dodonaea viscosa *var. *angustifolia* on the germ tube formation by *C. albicans*. Comparison of germ tube formation at 3.125, 1.56, and 0.78 mg/mL concentrations to the control is ∗, ∗∗, and ∗∗∗, respectively.

Subinhibitory concentrations of DVA extract significantly (*P* < 0.01) inhibited the germ tube formation by *C. albicans* at all test concentrations compared to the control (Figure 1). The decrease in the germ tube formation was 85.36, 61.91, and 26.27% at concentrations of 3.125, 1.56, and 0.78 mg/mL of plant extracts, respectively. Increased concentration of plant extract increased the inhibitory effect on the germ tube formation. All three strains behaved similarly. Acetone had no effect on germ tube formation.

### 3.2. Effect on the Biofilm Formation

**Figure 2:**
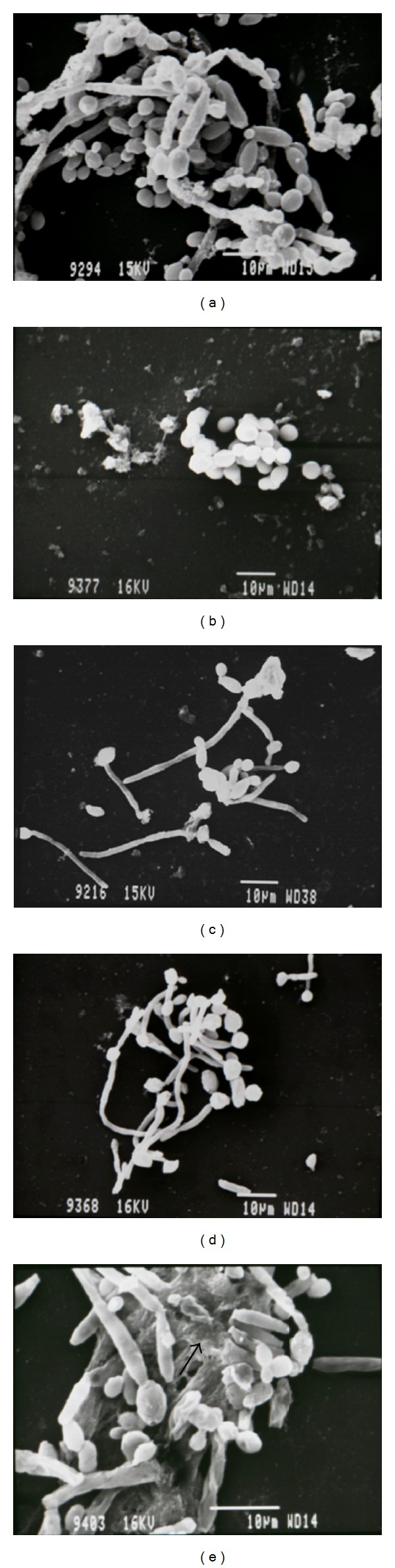
SEM showing the effect of *Dodonaea viscosa *var. *angustifolia* on the biofilm formation by *C. albicans*. (a) Control—2000x magnification, (b) extract 3.125 mg/mL—1500x magnification, (c) extract 1.56 mg/mL—1500x magnification, (d) extract 0.78 mg/mL—1500x magnification, and (e) control with extracellular polymeric matrix (arrow)—2700x magnification.

Subinhibitory concentrations of DVA extract reduced the biofilm and hyphae formation on plastic cover slips (Figure 2). The effect was concentration dependent. As the concentrations increased, biofilm formation and hyphae formation decreased. All three strains behaved similarly. Biofilms in the control tests had cells with the usual rounded morphology and bound together with extracellular matrix. Biofilms grown in the presence of plant extract had no extracellular matrix and some of the cells had abnormal morphology and were visually damaged.

### 3.3. TEM of Planktonic Yeast Cells

**Figure 3:**
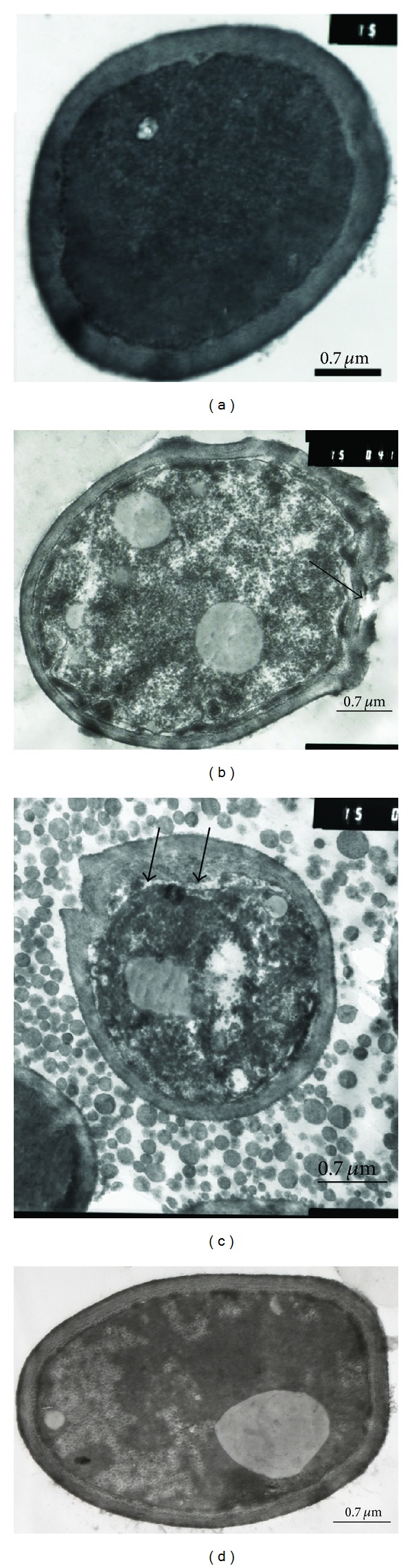
TEM showing the effect of *Dodonaea viscosa *var. *angustifolia* on the *C*.* albicans* cells. (a) Control, (b) 3.125 mg/mL, (c) 1.56 mg/mL, and (d) 0.78 mg/mL showing change in the cell density and damaged cell wall (arrow in (b)), undulation and detachment of cell membrane from cell wall (arrow in (c)).

The subinhibitory concentrations of crude plant extract visually damaged the cell wall and caused undulation in the cell membrane (Figure 3). The cytoplasm of the control cell was denser compared to the cells treated with plant extract. The plant extract also rendered the cytoplasm granular in appearance and large vacuoles were also present suggesting that cell permeability was affected.

## 4. Discussion

Commensal *C. albicans* yeast cells usually exist within biofilms in the oral cavity, vagina, on prostheses, and medical implants. Cells within biofilms have unique phenotypic characteristics compared to planktonic cells [16] and biofilm formation is considered a virulence factor. Microbes growing as biofilm are highly resistant to antimicrobial agents [17]. Plant such as *Cassia spectabilis* and *Scutellaria baicalensis* have been shown to inhibit biofilm formation by *C. albicans* [18, 19]. A previous study demonstrated that DVA extract inhibits adherence of *C. albicans* to host cells [20], and the present study shows that it also inhibits adherence to inert material. This has major implications for denture wearers and patients with head and neck cancers who wear prostheses. The oral cavities of these patients are generally colonized with Candida [21] and often these organisms are associated with the use of prostheses [22]. The use of this plant extract as a mouthrinse would reduce the adherence of *C. albicans* to prostheses and reduce the chances of infection. In addition, adherent cells would not be able to produce hyphae, which is absolutely necessary for the pathogenesis. 

Our results also showed that the crude plant extract of DVA can inhibit germ tube formation by planktonic cells of *C. albicans* which are responsible for host tissue penetration in the disease process. This correlated with studies done with essential oil from *Coriandrum sativum* (coriander) and *Ocimum gratissimum* (Alfavaca) where subinhibitory concentrations inhibited germ tube formation of *C. albicans *[23, 24]. This effect may have been due to cell wall damage because the biology of hyphal formation involves a complex process including growth of the hyphal tube requiring cell wall generation. Our results showed that DVA damaged the cell wall which may have caused changes in the cellular content. Similar results were found by Sangetha et al. (2009) who investigated the antimicrobial activity of the medicinal plant, *Cassia spectabilis* against *C. albicans* at an ultrastructural level, and found notable alterations in the cell wall and membrane [19]. 

Phytosterols and tannins are known to cause damage to fungal cells [25, 26]. Volatile oils and triterpenoids have been found in DVA [27, 28]. Our previous study has also shown presence of tannins such as methyl 4-O-methyl-á-D-xylopyranoside in DVA [29]. Plant derived tannins affects lipids of the cell membrane [30], similar to antifungal drugs such as azoles. In addition, terpenoids cause changes in the *C. albicans* cell wall thickness, cell morphology, shape, and alter adhesion [26, 31]. Presence of both chemicals in DVA (phytosterol and tannins) may have caused the extensive cellular damage, including that to the cell wall. Cell wall damage may have led to some dysfunction affecting cell division, budding, and hyphae formation. Similarly, it may have also affected the adherence of this organism to surfaces such as epithelial cells, acrylic dentures, and prostheses, which has been previously reported [20]. Beneficial effects at low concentrations are ideal because it is difficult to maintain high concentrations of therapeutic agent in the mouth due to the constant flow of saliva. New antifungal agents targeting the yeast cell wall will be of great value in the treatment of oral candidiasis, especially since drug resistance has emerged and existing drugs such as amphotericin B target the cell membrane which increases toxicity.

Our findings also showed that the extracellular polymeric matrix was present in the biofilm grown without the plant extract but was absent in biofilms which were grown in the presence of plant extract. The extracellular polymeric matrix may restrict the penetration of antifungal drugs rendering treatment ineffective. Since the safety of this plant has been established [32, 33], use of this plant extract in conjunction with an antifungal drug would be ideal as the extracellular polymeric material would be absent and the few *C. albicans* cells still present will have unrestricted exposure to the antifungal drug. Use of traditional medicine with conventional medicine as an additional therapeutic agent has been found to have a synergistic effect with low MIC values [34]. 

In conclusion, the plant extract of DVA reduces hyphae and biofilm formation in *C. albicans* which could be a result of damage to the cell wall. Hyphal and biofilm formation are important interrelated virulence factors in the pathogenesis of candidal infections. Therefore this plant has therapeutic potential and future research is required to establish the chemical constituents responsible for these beneficial effects.
